# A Hierarchical Prediction Method for Pedestrian Head Injury in Intelligent Vehicle with Combined Active and Passive Safety System

**DOI:** 10.3390/biomimetics9030124

**Published:** 2024-02-21

**Authors:** Liangliang Shi, Honghao Zhang, Lintao Wu, Yu Liu, Kuo Cheng, Yong Han, Danqi Wang

**Affiliations:** 1 State Key Laboratory of Vehicle NVH and Safety Technology, China Automotive Engineering Research Institute Co., Ltd., Chongqing 401122, China; shiliangliang@caeri.com.cn (L.S.); honghao_zhang@sdu.edu.cn (H.Z.); liuyu@caeri.com.cn (Y.L.); chengkuo@caeri.com.cn (K.C.); 2 Key Laboratory of High Efficiency and Clean Mechanical Manufacture (Ministry of Education), School of Mechanical Engineering, Shandong University, Jinan 250061, China; 3 College of Automotive and Mechanical Engineering, Changsha University of Science & Technology, Changsha 410114, China; wlt052410@163.com; 4 School of Mechanical and Automotive Engineering, Xiamen University of Technology, Xiamen 361024, China; yonghan@xmut.edu.cn

**Keywords:** active and passive safety, intelligent car, engine hood airbag, head injury

## Abstract

With the development of intelligent vehicle technology, the probability of road traffic accidents occurring has been effectively reduced to a certain extent. However, there is still insufficient research on head injuries in human vehicle collisions, making it impossible to effectively predict pedestrian head injuries in accidents. To study the efficacy of a combined active and passive safety system on pedestrian head protection through the combined effect of the exterior airbag and the braking control systems of an intelligent vehicle, a “vehicle–pedestrian” interaction system is constructed in this study and is verified by real collision cases. On this basis, a combined active and passive system database is developed to analyze the cross-influence of the engine hood airbag and the vehicle braking curve parameters on pedestrian HIC (head injury criterion). Meanwhile, a hierarchy design strategy for a combined active and passive system is proposed, and a rapid prediction of HIC is achieved via the establishment of a fitting equation for each grading. The results show that the exterior airbag can effectively protect the pedestrian’s head, prevent the collision between the pedestrian’s head and the vehicle front structure, and reduce the HIC. The braking parameter *H*_2_ is significantly correlated with head injury, and when *H*_2_ is less than 1.8, the HIC value is less than 1000 in nearly 90% of cases. The hierarchy design strategy and HIC prediction method of the combined active and passive system proposed in this paper can provide a theoretical basis for rapid selection and parameter design.

## 1. Introduction

China’s road traffic environment is complex, and the issue of pedestrian–vehicle mixed traffic is serious, resulting in a high frequency of pedestrian–vehicle collisions. With the rapid development of modern perception, decision-making, and communication technology, intelligent driving technology has emerged in response. The emergence of intelligent driving technology can reduce collision accidents caused by improper driver operation to a certain extent; however, for pedestrians, a strong uncertainty factor in the transportation system with respect to pedestrian–vehicle collision is hard to completely avoid, and the mortality rate of pedestrians remains very high. Therefore, the mechanisms of pedestrian injury and collision prevention measures remain research hotspots in the field of automobile safety [1,2,3].

According to relevant data released by the National Bureau of Statistics, there were a total of 256,409 road traffic accidents in China in 2022, with 60,676 deaths and a mortality rate of 23.66% [4]. Among them, pedestrian injuries mainly come from frontal collisions with cars. The head and legs are the areas with the most injuries, and 64% of pedestrians die from head injuries [5].

Currently, intelligent vehicles use a combined active and passive system in an attempt to minimize pedestrian injuries in collisions. The active safety systems include autonomous emergency braking (AEB), autonomous emergency steering (AES), etc. [6,7,8]. Fu et al. [9] proposed a deep reinforcement learning (DRL)-based autonomous braking decision-making strategy in emergency situations by analyzing the vehicle lane changing process and braking process in detail, taking into account balance among the three key influencing factors of efficiency, accuracy, and passenger comfort to improve driving safety. Alsuwian [10] proposed an advanced sensor fusion based on an emergency braking system (EBS) that automatically identified possible collisions and activated the vehicle braking system. An emergency steering-collision-avoidance method based on safety distance as an index was proposed by Chen et al. [11], which calculates the required safety distance encountering a stationary or abruptly decelerating vehicle at different speeds via slip angles and lateral force tire models. In order to improve the braking system performance of an intelligent vehicle to avoid pedestrian collisions, Yang et al. [12] proposed an automatic emergency braking collision avoidance (AEB-P) model based on a fuzzy neural network (FNN) and a genetic algorithm (GA).

Active safety methods are generally analyzed from the perspective of collision avoidance, while passive safety methods are analyzed from the perspective of reducing the injuries caused by collisions, which usually involve vehicle structure, the restraint system, material properties, etc. [13,14]. Li et al. [15] proposed a data-driven design method to optimize the engine hood through a machine learning model in order to achieve the lightweighting of the engine hood and to improve pedestrian safety. Mizuno et al. [16] conducted a multi-body simulation analysis of different vehicle types and pedestrian body sizes and found that the decrease in pedestrian–vehicle re-contact was essential to reduce ground contact injury. The severity of pedestrian injury was not only related to vehicle speed but also to pedestrian sign, vehicle front parameter, and each participant motion involved in the collision [17,18,19,20,21,22]. Researchers have proposed to study the correlation between collision velocity, vehicle front parameter, pedestrian sign, and participant motion characteristics to injuries [23,24], which combines with intelligent driving technology to better understand the injury mechanism and collision mechanism of pedestrians in collisions, as well as to minimize the probability of collision and the risk of injury as much as possible via active vehicle braking measures [25]. Zou et al. [26,27,28] proposed a control vehicle braking mode to reduce pedestrian–ground contact injuries in collisions by controlling the time of pedestrian landing. Li et al. [29] used adaptive model predictive control (AMPC) to combine four-wheel steering, active rear-wheel steering, and differential braking to propose an integrated steering- and braking-based collision avoidance strategy for emergency situations in autonomous vehicles.

In recent years, researchers have also conducted extensive research on damage prediction. Shouhei K conducted collision simulation experiments using finite element Sedan models and MADYMO pedestrian models to generate image datasets and used training datasets for deep learning to determine the impact of pedestrian physical differences in predicting head injuries in vehicle pedestrian accidents [30]. Ashique E used fracture modeling techniques to simulate a frontal collision between a vehicle and a rigid wall and used a human body model to study fractures and tears [31]. Pushpender P used multi-body software and finite element software to reconstruct human vehicle collision accidents, obtaining relevant collision conditions such as vehicle speed and pedestrian position through multi-body simulation and using them as initial conditions for a finite element simulation comparing the damage prediction ability of the THUMS finite element human body model in real human vehicle collisions [32]. Won conducted multiple low-speed rear end collision tests and used machine learning-derived multiple linear regression to predict the motion of the impacted vehicle and the neck injury standards of passengers under test conditions to evaluate the likelihood of neck injury in low-speed traffic accidents [33].

Most of the existing studies have qualitatively analyzed the impact of a combined active and passive system on pedestrian injuries in different collisions or designed the active and passive system separately to reduce pedestrian injuries, while fewer studies have been conducted on the quantitative evaluation and prediction of pedestrian injuries in intelligent vehicles with a combined active and passive system [34,35]. Focusing on the microscopic transportation system of intelligent vehicle–pedestrian collision, this paper constructs an interaction model containing vehicle braking control, engine hood airbags, pedestrian head injury, etc., to explore the hierarchical prediction method of pedestrian injury in the combined effect of vehicle braking control and engine hood airbags and to provide a theoretical basis for the development and application of an intelligent vehicle braking strategy and engine hood airbag [36].

The contributions of this study include the following: (1) An interactive model containing a vehicle brake system, engine hood airbag, pedestrian head injury, and other elements is constructed; the key parameters of the vehicle braking curve are extracted; and a database of pedestrian head injury is established via a combined active and passive system. (2) The influence of the combined effect of an active and passive system in an intelligent vehicle on pedestrian head injury is quantitatively analyzed, and the relevant conclusions are obtained to guide the design of a braking system and engine hood airbag. (3) A classification prediction method of pedestrian head injury is constructed to provide a theoretical basis for the real-time control of an intelligent vehicle braking system.

**Figure 1:**
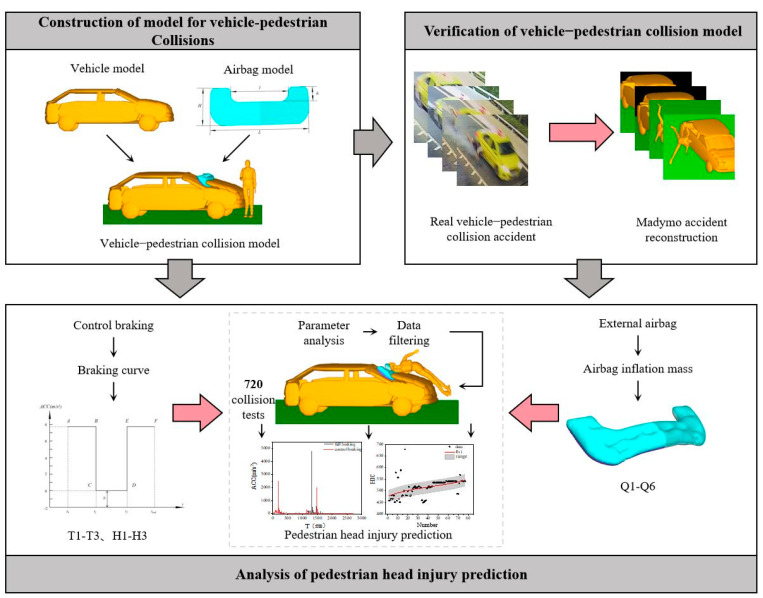
Conceptual framework.

This study is structure as follows: Chapter II: Modeling and validation of pedestrian–vehicle interaction with active and passive system; Chapter III: Constructing a database of pedestrian head injuries in intelligent vehicles with a combined active and passive system for subsequent research on injury parameter analysis; Chapter IV: Analyzing the cross-mixing effects of exterior airbags and vehicle braking curve parameters on pedestrian head injuries and obtaining conclusions that can guide the active and passive synergistic design of airbags and braking curves in intelligent vehicles; Chapter V: Through the results of parameter analysis, different categories of airbags are classified to establish the pedestrian head injury prediction function, which realizes the rapid prediction of the severity of pedestrian head injuries in collisions. The overall research framework is shown in Figure 1.

This article studies the combined effect of active and passive safety systems on pedestrian head protection under the combined action of external safety airbags and control braking in intelligent vehicles. Taking the combination of safety airbags and control braking as an innovative point, the combined effect on pedestrian head injury is comprehensively considered. The aim is to reduce pedestrian head injury in traffic accidents through the combination of active and passive methods, providing a certain theoretical basis for the development of intelligent vehicles in the future.

## 2. Modeling and Validation of Intelligent Vehicle–Pedestrian Interaction Systems

### 2.1. Intelligent Vehicle–Pedestrian Interaction System with a Combined Active and Passive System

**Figure 2:**
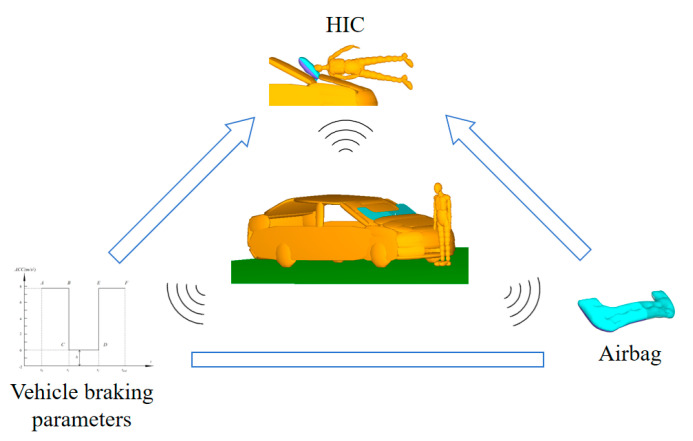
Vehicle–pedestrian interaction system.

This study aims to explore the influencing factors of pedestrian head injury based on an intelligent vehicle with a combined active and passive system, and the defined system in this paper contains key elements such as vehicle braking control curves, engine hood airbags, vehicles, pedestrians, and the head injury indicators used to evaluate the extent of pedestrian injuries in collisions (Figure 2).

**Figure 3:**
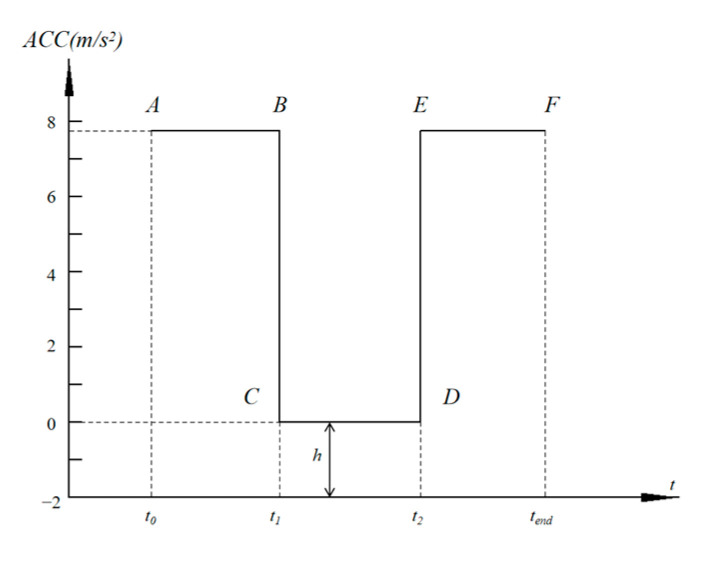
Vehicle braking control curve.

It has been found that changing the vehicle braking mode can impact the probability of pedestrian landing injury and pedestrian head injury in a vehicle–pedestrian collisions [37,38]. Considering the realizability of intelligent vehicle braking curve regulation, the braking curve is expressed in a three-step method, where *t*_0_ is the moment of pedestrian–vehicle collision; *t*_1_ is the first contact time of the pedestrian’s head with the vehicle hood; *t*_2_ is the complete braking time of the vehicle to prevent running over the pedestrian and causing secondary injury; and *t_end_* is the stopping motion of vehicle (Figure 3). The period from *t*_0_ to *t*_1_ is defined as *T*_1_*, t*_1_ to *t*_2_ is defined as *T*_2_, *t*_2_ to *t_end_* is defined as *T*_3_, and the braking deceleration values for each period are defined as *H*_1_ (braking value of *T*_1_ segment), *H*_2_ (braking value of *T*_2_ segment), and *H*_3_ (braking value of *T*_3_ segment), respectively.

The effect of engine hood airbags on pedestrian head protection is affected by factors such as inflation time, inflation speed, inflation mass, and airbag shape. However, in order to study the effect of the airbag, it is required that the airbag fully deploys when the contact of the pedestrian’s head and vehicle occurs; therefore, the time and efficiency of airbag inflation are not explored. The inflation mass in this system is defined as the adjustable parameter of the airbag. The design scheme is as follows: on the basis of determining the airbag shape, the original inflated mass of the airbag is defined as the standard and changes by percentage. In order to prevent the airbag providing no buffering effect by small inflation mass and surface stiffness by excessive inflatable mass, the airbag inflation mass is divided into 6 grades (the parameters inflation mass of *Q*_1_–*Q*_6_) with 10% as a step, which is successively reduced to 70% and increased to 130%.

Studies have found that pedestrian head injuries are more likely to lead to permanent injury, and serious cases can lead to pedestrian death. Therefore, the pedestrian HIC is used as an evaluation index by which to evaluate the severity of pedestrian injury in the system, and the mathematical definition is as follows:

$$HIC={\left\{\left(t_{2}-t_{1}\right){\left[\frac{1}{t_{2}-t_{1}}\int_{t_{1}}^{t_{2}}\;\left(\frac{a(t)}{g}\right)dt\right]}^{2.5}\right\}}_{max}$$

a(t): The resultant head acceleration(m/s^2^).

T_1_ and t_2_ (s): The moments when HIC achieves its maximum value, respectively.

G: Gravitational acceleration, 9.81 m/s^2^.

### 2.2. System Modeling

**Figure 4:**
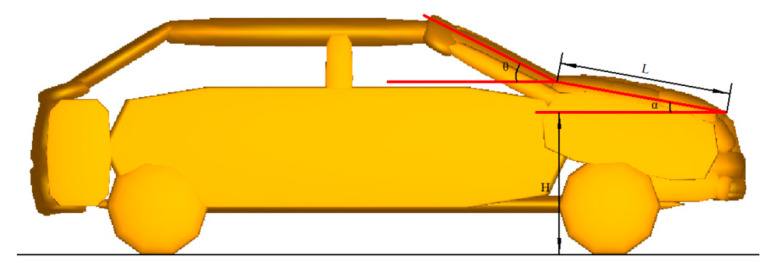
Model of the vehicle–pedestrian multi-body interaction system.

Based on the future mobile traffic accident scenario study (FASS), one vehicle–pedestrian collision is selected for accident reconstruction and parameter research. First, PC-Crash is used to restore the accident scene and obtain the dynamic parameters of vehicles and pedestrians. Second, the corresponding vehicle–pedestrian multi-body model is constructed based on the reconstruction result of PC-Crash, and the multi-body models of the vehicle braking curve and engine hood airbag are integrated to establish the vehicle–pedestrian interaction system model with a combined active and passive system. The Hybrid III 50th percentile male dummy model is used as the pedestrian in the system. Vehicle front structures, including the bumper, hood, fender, windshield, and wheels, directly interact with pedestrians. The hood leading edge height (H) is 0.75 m, the hood length (L) is 1.16 m, the inclination angle (*α*) of vehicle hood is 10.43°, and the inclination angle (*θ*) of windshield is 10.43° (Figure 4).

**Figure 5:**
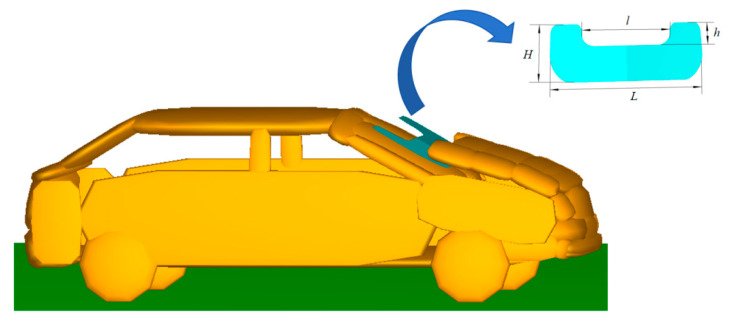
Structure of the engine hood and location of parts.

The engine hood airbag in the system is “U” shaped and installed in the hood below the rear edge, in which the length (L) and width H of the airbag after the expansion is 150 cm and 60 cm, respectively; the length (L) of the upper edge of the airbag is 90 cm; and the height (H) of the upper edge is 20 cm, as shown in Figure 5. The ignition of the airbag is set to be the moment *t*_0_ when pedestrian–vehicle contact occurs. The functions of airbag mass flow and temperature are correlation functions of MADYMO airbag model.

### 2.3. Model Validation

**Figure 6:**
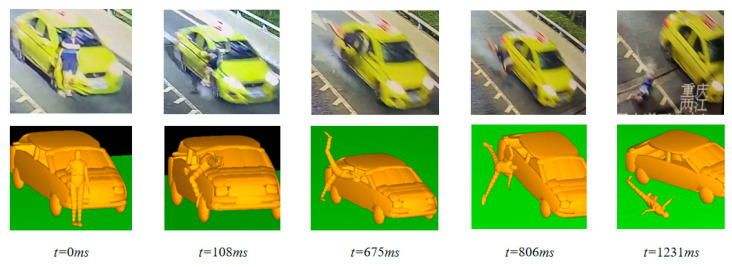
Verification of PC-Crash vs. MADYMO models.

**Figure 7:**
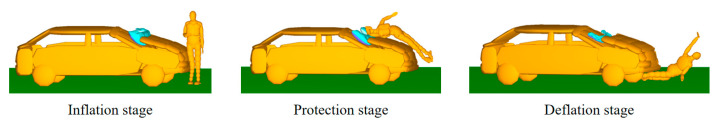
Verification of airbag inflation and deflation.

The model validation is divided into two steps. The first step is to compare and analyze the vehicle–pedestrian collision kinematic pattern via the real accident video and the MADYMO model, as shown in Figure 6. The model kinematic process is basically consistent with the accident video, which meets the analysis requirement. In the second step, based on the completion of the kinematic verification, the FE model of the exterior airbag is constructed and coupled with the multi-body model, which is then run in the MADYMO model to observe whether the airbag can be normally ignited and exploded, inflated, and deflated, etc. The computational results are shown in Figure 7, indicating the good operation of the engine hood airbag. The verification results show that the model can be used in the subsequent simulation of the active and passive systems of intelligent vehicles.

## 3. Construction of Intelligent Vehicle Database with a Combined Active and Passive System

### 3.1. Data Sample Screening

**Figure 8:**
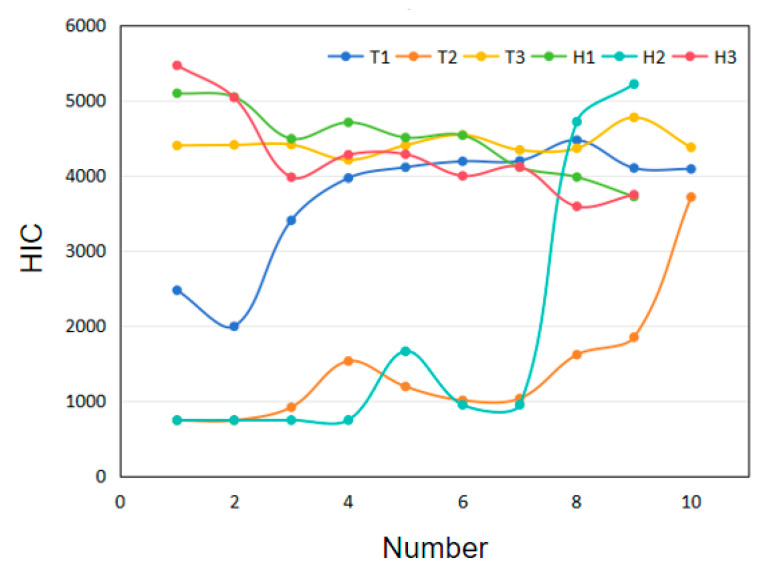
Parameter analysis of HIC variation.

In this paper, based on the intelligent vehicle model with a combined active and passive system, a database containing braking curve parameters, engine hood airbag parameters, and pedestrian HIC is established. Control variable analysis is used to analyze the data of the six vehicle braking curve parameters in Figure 3, and the parameters with greater influence on the system are screened out as design variables for subsequent research. The three moments (Ts) are defined as varying within 200 ms and H as vary within 0–7.8 ms. The pedestrian HIC value is calculated by the model shown in Figure 8. In this Figure, the horizontal coordinate is the data number, and the vertical coordinate is the HIC value, and each curve represents the change in the HIC value for the 10 levels of this parameter.

As can be seen in Figure 8, fluctuations in *T*_3_, *H*_1_, and *H*_3_ have a relatively small effect on HIC, whereas *T*_1_, *T*_2_, and *H*_2_ have a relatively large effect on HIC. *T*_3_ and *H*_3_ causing a small fluctuation is due to the EF section of the braking curve being in the late stage of the collision and the pedestrian–vehicle collision having ended, so the impact is smaller. *H*_1_ is the braking value of the AB section of the braking curve, which is at the beginning of the collision. As the relative pedestrian–vehicle collision position is very close during the simulation, the time from the beginning of the experiment calculation to the first pedestrian–vehicle contact is relatively short, and the vehicle braking time is relatively short, which will have a small impact on the collision speed, so the impact of *H*_1_ on the HIC is relatively small. By analyzing the braking parameters, three braking parameters, *T*_1_, *T*_2_, and *H*_2_, are selected for subsequent database construction in this study.

### 3.2. Database Construction

In this paper, ordinary passenger cars and normal-sized 50t- percentile male dummies are selected as the study subjects. According to the traffic accident data, there is a large percentage of pedestrian–vehicle collisions with speeds lower than 45 km/h (about 80%), so this study analyzes collisions at speeds of 40 km/h. The braking parameter *T*_1_ is selected as 85–185 ms, with an increase of 25 ms intervals (five groups in total); the braking parameter *T*_2_ is selected as 100–200 ms with an increase of 20 ms intervals (six groups in total); and the braking parameter *H*_2_ is selected as 0 m/s^2^ and 1.8–5.8 m/s^2^ with an increase of 2 m/s^2^ intervals (four groups in total), respectively. An inflation mass of 70–130% (six groups) for airbags is selected for the study. The experimental design is carried out through the orthogonal experiment method. The 720 sets of experimental results are obtained to analysis the relationship between pedestrian HIC value and the active and passive systems of an intelligent vehicle. The samples in the database are the basis for the parameter analysis of an intelligent vehicle with a combined active and passive system.

## 4. Parameter Analysis

### 4.1. Parameter Analysis of Engine Hood Airbag

**Figure 9:**
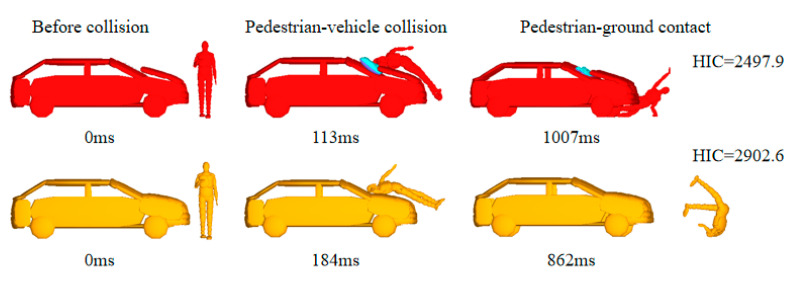
Collision comparison with and without airbags.

**Figure 10:**
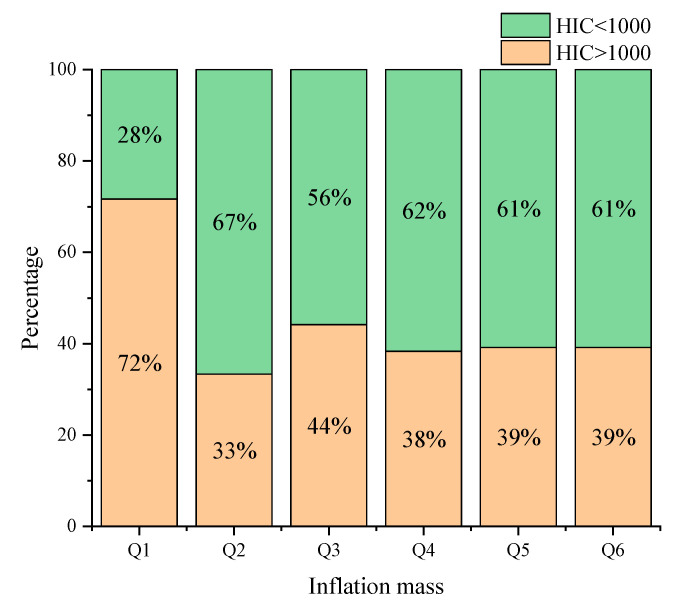
HIC percentage at different airbag inflation masses.

In the case of the same braking curve parameters, the comparison of the pedestrian–vehicle interaction model calculations under the combined effect of active and passive systems with and without an engine hood airbag is shown in Figure 9, in which the first pedestrian–vehicle collision is essentially the same if the other collision parameter variables are kept consistent. In the event of a collision, vehicles with engine hood airbags have some crash cushioning as the pedestrian head comes into contact with the airbag, whereas without engine hood airbags, this causes greater head injury when the pedestrian head collides directly with the windshield/hood during a collision. The HIC value caused by the vehicle equipped with airbags is 756.92, and not equipped with airbags, the value is 1790.8, which indicates that the presence of engine hood airbags effectively reduces the HIC value, as shown in Figure 10. In addition, the engine hood is popped up when the airbag is inflated in a vehicle equipped with airbags, leaving a certain buffer space for the pedestrian–hood collision and playing the role of energy absorption. However, due to the popping up of the airbag and engine hood, the height of vehicle front end changed, resulting in an increase in the throwing distance of the pedestrian after the collision, which had a certain impact on the pedestrian landing injury.

Varying the airbag inflation mass and organizing and analyzing the experimental data revealed that about 72% of the simulated cases had HIC values exceeding 1000 when the airbag inflation mass was 70%. At this time, the engine hood airbag cannot effectively protect the pedestrian head in pedestrian–vehicle collision. When the airbag inflation mass is other variables, the HIC value is below 1000 in nearly 60% of the simulation cases, which indicates that the existence of the engine hood airbag can provide better protection for the pedestrian head. With the increase in the restarting mass of the engine hood airbag, the HIC value has no obvious trend, indicating that when the inflation mass of the engine hood airbag is within a reasonable range, the pedestrian head injury is determined by the collision speed, position, front-end structure and stiffness, and the vehicle braking process.

### 4.2. Vehicle Braking Curve Parameter Analysis

**Figure 11:**
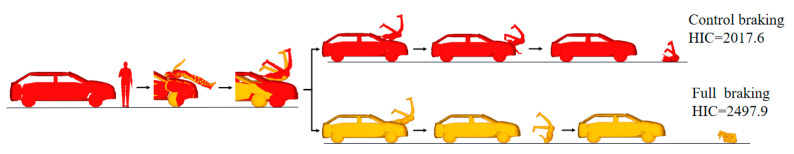
Collision process of different braking methods.

In a pedestrian–vehicle collision, the intelligent vehicle decelerates by controlling the braking method, which can change the motion state of the pedestrian after contact with the vehicle. In order to analyze the effect of the vehicle braking process on the pedestrian HIC value, the process of the pedestrian’s motion is compared with the vehicle-controlled braking and the vehicle’s full braking without the engine hood airbag, as shown in Figure 11. It can be seen that at the same moment, different braking modes result in different pedestrian motion states. In the initial phase, the pedestrian’s lower limbs come into contact with the vehicle bumper and the upper body rotates around the collision point, causing the head to collide with the windshield/hood. This phase is consistent with the pedestrian’s course of motion as both are applying full braking. As both take full braking, the course of motion at this phase is consistent with the course of pedestrian motion. When the pedestrian head and vehicle contact is separated, the brake value will be reduced to 0 m/s^2^ in the controlled braking mode, and then the vehicle continues to keep moving forward, causing the pedestrian and the front end of the vehicle contact again so as to act as a certain buffer for the pedestrian landing. In the case of the complete braking mode, the pedestrian will be directly on the ground, which will result in a greater injury. With the application of a full braking, the pedestrian will land directly on the ground, and this will cause severe injury.

**Figure 12:**
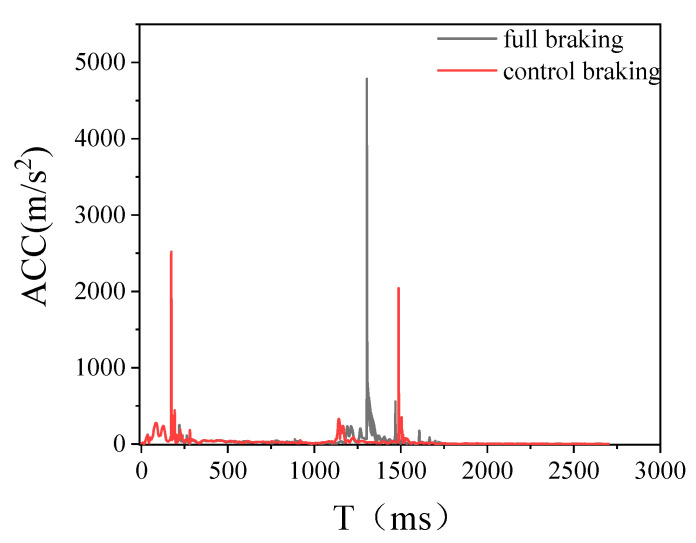
Comparison of the gravity acceleration of the head under different braking methods.

The head acceleration curves for the two braking methods are shown in Figure 12. The peak of head acceleration caused by the full braking method is higher than that caused by the controlled braking method; thus, the head injury is correspondingly worse. Controlling the braking methods can effectively change the pedestrian landing state, so the second contact motion of pedestrians and vehicles is consistent, to a certain extent, with the vehicle motion, reducing the kinetic energy brought about by the collision and thus effectively reducing the pedestrian landing injury.

The HIC values obtained from the crash simulation were screened and the correlation between the braking curve sensitive parameters *T*_1_, *T*_2_, and *H*_2_ and HIC were analyzed using Pearson’s correlation, as shown in Table 1. *T*_2_ and *H*_2_ were significantly correlated with HIC values, and *T*_1_ was relatively correlated. Therefore, when the braking curve parameters are optimized, priority should be given to optimizing *T*_2_ and *H*_2_ and then *T*_1_. Among them, *T*_1_ and *T*_2_ are negatively correlated with HIC value, and as *T*_1_ and *T*_2_ increase, the HIC value of head injury decreases accordingly. When *T*_1_ and *T*_2_ have greater values, most of the HIC values caused are below the threshold value of 1000, while when *T*_1_ and *T*_2_ have lesser values, the HIC values exceed the threshold value of 1000. *H*_2_ and HIC values are positively correlated; i.e., *H*_2_ increases the HIC value accordingly. When *H*_2_ has a lesser value, the majority of the HIC values caused by the collisions are below the threshold value of 1000, with only 10% of cases having an HIC above 1000.

### 4.3. Parametric Analysis of a Combined Active and Passive System

**Figure 13:**
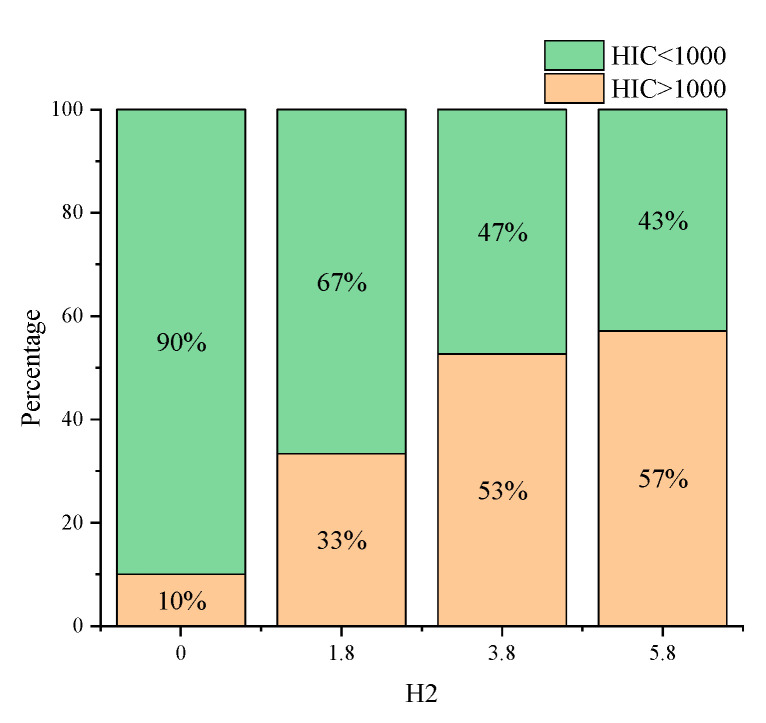
HIC value corresponding to different *H*_2_ values for cases with 80–130% inflation mass.

In order to analyze the effect of the system parameters on pedestrian head injuries in the combined effect of vehicle braking control and engine hood airbags, cross-classification analyses are performed in this chapter for airbag parameters and braking curve parameters, respectively. By filtering the cases with an airbag inflation mass of 70%, the cases with an inflation mass of 80–130% were categorized and analyzed by the vehicle braking parameter *H*_2_. As shown in Figure 13, when the braking parameter *H*_2_ has a lesser value, most of the pedestrian HIC values caused by the collision are below the threshold value of 1000, and only 10% of the cases with HIC exceed 1000, at which time the braking curve is obviously concave. Conversely, when the vehicle braking parameter *H*_2_ has other braking values, the overall collision injury is relatively large and shows an increasing trend as the braking value of *H*_2_ increases.

In order to obtain the relative optimal braking curves, the braking value *H_2_* was used to classify and analyze the effect of other braking parameters (*T*_1_, *T*_3_) on the pedestrian HIC values at different *H*_2_ values. Through the screening of the collision data, we can find that when *H*_2_ is less than 1.8, the pedestrian HIC value conducted in collision simulation experiments is smaller, and the protection effect of the pedestrian head injury is relatively optimal and is less affected by other collision parameters. As *H*_2_ is greater than 1.8, the pedestrian HIC shows different trends influenced by different airbag inflation masses as well as other braking curve parameters. To further analyze the parameter characteristics of obtaining lower HIC, the simulation experimental data were screened as shown in Table 2. When *Q*_1_ is selected as the inflation mass value and *H*_2_ is more than 1.8, *T*_1_ is taken as 135–185 ms and *T*_2_ is taken as 160–200 ms, and the HIC values in the collision simulation experiments are less than 1000 in 85% of cases. When *Q_2_* is selected as the inflation mass value and *H*_2_ is more than 1.8, *T*_1_ is taken as 160–185 ms and *T*_2_ is taken as 160–200 ms, and the HIC value in the collision simulation experiments is less than 1000 in 78% of cases. Several other inflated gas mass cases are shown in Table 2. Other inflation mass outcomes are shown in Table 2.

**Figure 14:**
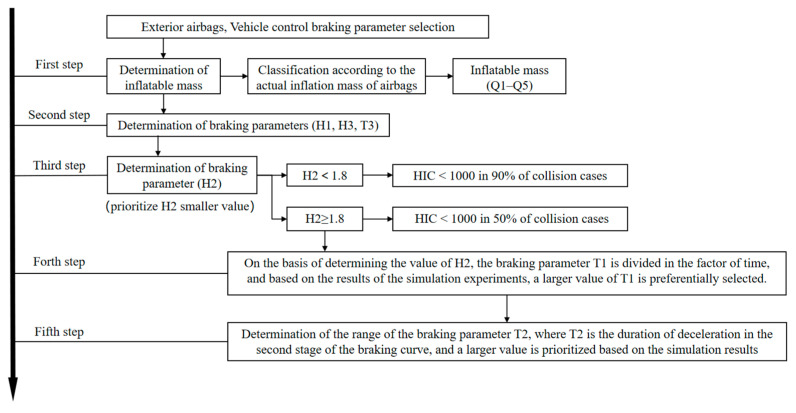
Parameter hierarchy design scheme for active and passive systems.

Based on Table 2, this paper proposes a parametric hierarchy design for an active–passive system, and the basic design idea is shown in Figure 14. The design steps are as follows.

First step: Exterior airbags, vehicle control braking parameter selection, determination of inflatable mass, classification according to the actual inflation mass of airbags, and inflatable mass (*Q*_1_–*Q*_5_).

Second step: Determination of braking parameters (*H*_1_, *H*_3_, *T*_3_).

Third step: Determination of braking parameter (*H*_2_), prioritize smaller *H*_2_ value; *H*_2_ < 1.8, HIC < 1000 in 90% of collision cases; *H*_2_ ≥ 1.8, and HIC < 1000 in 50% of collision cases.

Fourth step: On the basis of determining the value of *H*_2_, the braking parameter *T*_1_ is divided in the factor of time, and based on the results of the simulation experiments, a larger value of *T*_1_ is preferentially selected.

Fifth step: Determination of the range of the braking parameter *T*_2_, where *T*_2_ is the duration of deceleration in the second stage of the braking curve, and a larger value is prioritized based on the simulation results.

The airbag and braking parameters are selected and processed to lay the foundation for the subsequent prediction of pedestrian head injury through the above steps.

## 5. Prediction Classification Method of Pedestrian Head Injury

**Figure 15:**
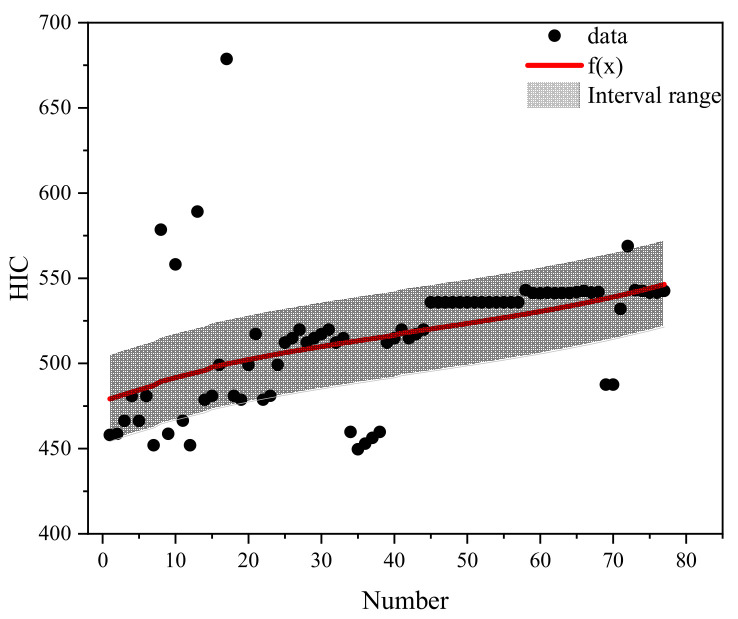
Fitted plot of inflated mass *Q*1.

**Figure 16:**
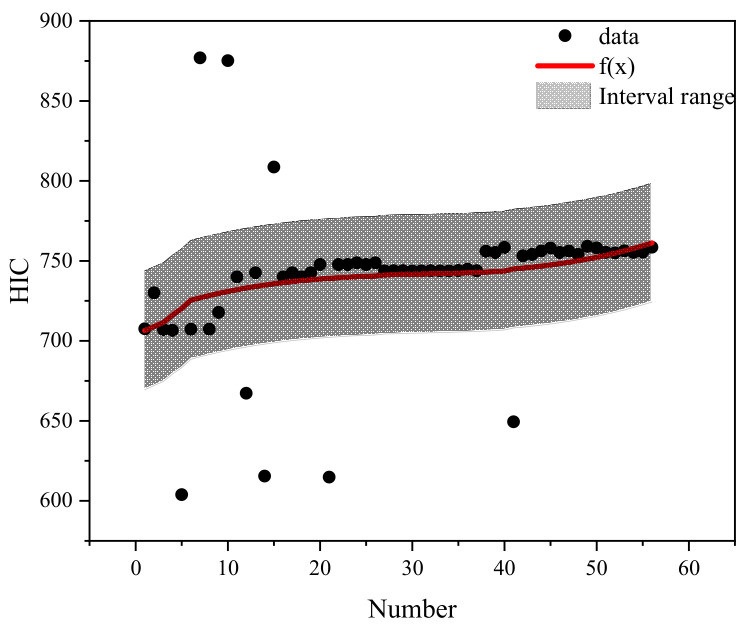
Fitted plot of inflated mass *Q*2.

**Figure 17:**
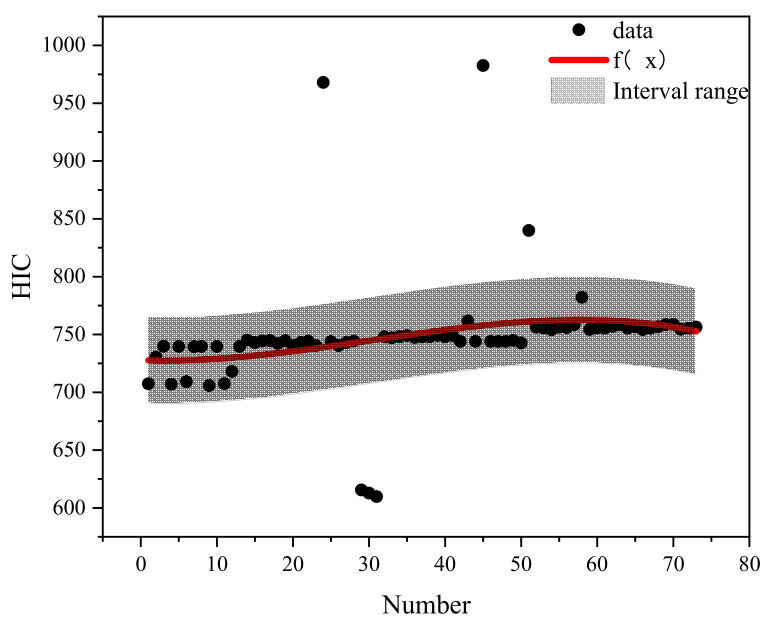
Fitted plot of inflated mass *Q*3.

**Figure 18:**
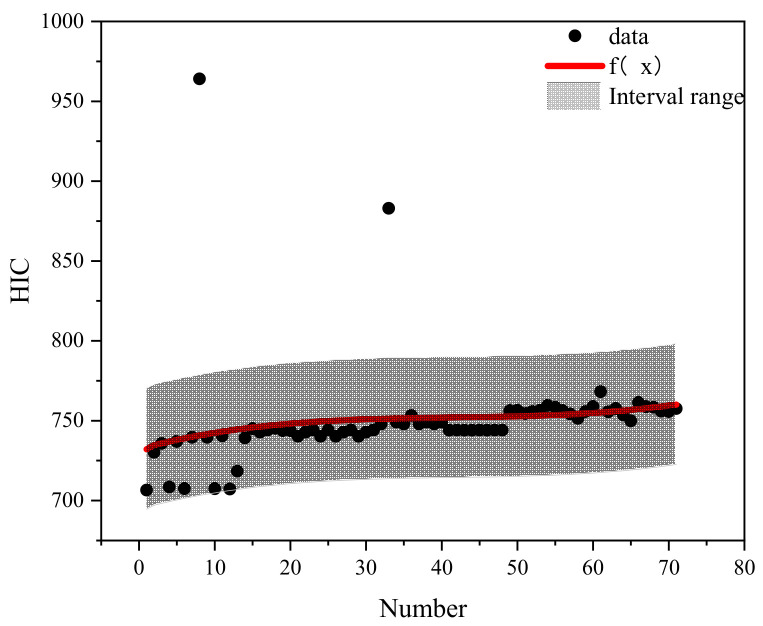
Fitted plot of inflated mass *Q*4.

**Figure 19:**
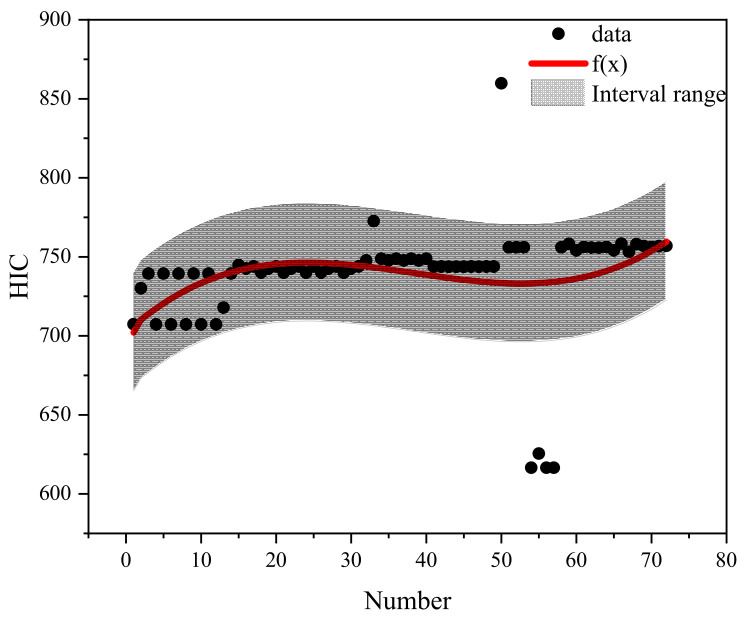
Fitted plot of inflated mass *Q*_5_.

Based on the different qualities of airbag inflation and categorization of the braking curve parameters, the case data were effectively filtered to conduct the regression analysis on pedestrian head injury under the protection of different qualities of airbag inflation. Different brake curve parameters by number were used as X-coordinates and HIC as Y-coordinates. The regression curves fitted to each airbag inflation mass are shown in Figure 15, Figure 16, Figure 17, Figure 18 and Figure 19. The airbag inflation can be determined first by the specific vehicle type and crash conditions in this study, and then the range of data points was estimated by the brake curve parameters, which were substituted into the corresponding regression curves for rapid predictive analysis of the HIC, as shown in Table 3.

This study is a numerical simulation experiment which has errors with real accident cases. Therefore, an error value of ±5% (gray area in the figure) is assigned to predict the severity of HIC for pedestrian head injuries when fitting the prediction function of HIC values. The airbag inflation mass of *Q*_1_ has the best protection effect, and the HIC value is lower than other inflation masses (Figure 15, Figure 16, Figure 17, Figure 18 and Figure 19); while in other inflation masses, the HIC value is below the threshold value of 1000, also providing good protection for the pedestrian head. And it can be observed that most collision data points are within the range of the interval, and very few collision data points are out of the range, probably due to the change in the braking parameters caused by the change in the pedestrian’s landing attitude, which increases/decreases the pedestrian head rotational acceleration and leads to a change in the HIC value. In future research, different exterior airbags should be used as the classification object, and the regression curve should be used as the prediction function for the rapid prediction of the pedestrian HIC value in collisions, which can provide a theoretical basis for the interpretation of the severity of pedestrian head injury.

## 6. Conclusions

The effects of engine hood airbag and vehicle control braking curve parameters on pedestrian HIC are effectively verified through simulation experiments in this study. By comparing the presence or absence of airbags, the braking curve was screened and analyzed to obtain the best parameters. The use of airbags reduces the HIC from 1790.8 to 756.92, and the braking curve has a significant effect on pedestrian–ground contact injuries. Through the employment of airbags and brake systems, the pedestrian head injury can be effectively reduced.

1. The engine hood airbag has a good protective effect on pedestrian head injury. In pedestrian–vehicle collisions, the engine hood airbag can protect the pedestrian’s head and avoid direct collision with the front windshield, engine hood, and other rigid parts, thereby effectively reducing the head injury. When the inflation mass of the engine hood airbag is less than 70%, nearly 70% of the cases in the simulation experiment have HIC more than 1000, and the airbag cannot effectively protect the pedestrian’s head in collisions.
2. Controlling the vehicle braking system process can effectively reduce the risk of pedestrian landing injury. A braking parameter *H*_2_ lower than 1.8 can effectively reduce HIC. Specifically, braking curve parameters such as *T*_1_, *T*_2_, and *H*_2_ have a high correlation with pedestrian head injury, in which *T*_1_ and *T*_2_ are negatively correlated with HIC, while *H*_2_ is positively correlated with HIC. Therefore, the larger values of *T*_1_ and *T*_2_ and the smaller value of *H*_2_ are preferred in selecting the braking curve value in priority.
3. In this paper, by analyzing the intelligent vehicle database with a combined active and passive system and the system parameters, a hierarchy design of an active and passive system is proposed, which can effectively reduce pedestrian HIC and avoid a large number of repetitive and erroneous simulation trials. The design sequence is as follows: the airbag inflation mass > the braking curve parameters *H*_1_, *H*_3_, and *T*_3_ parameters > braking parameter *H*_2_ > the braking parameter *T*_1_ > braking parameter *T*_2_.

The research is based on the complex traffic environment in Chinese society; the phenomenon of mixed traffic between people and vehicles is severe, and conflicts between people and vehicles occur frequently. In this paper, the active passive joint protection strategy of vehicles is studied with a view towards pedestrian head protection in traffic accident conflicts. The pedestrian head injury prediction method proposed in this paper solves the quantitative prediction problem of highly complex nonlinear systems and can provide a reference for the design and performance improvement of intelligent vehicle active and passive systems.

There are some limitations in this study: (1) The vehicle braking curve proposed in this study is mainly intended for the collision between a Sedan and pedestrian to reduce the pedestrian–ground contact injury. The analysis and outcome are unclear for other vehicle types, and there may even be a small increase in pedestrian head injuries in the simulation trial. (2) It is necessary to further improve the effectiveness of engineering application parameters in the future in order to obtain more detailed hierarchy design criteria. (3) The simulation model built in this paper is a restoration of the accident case, and the subsequent database construction and parameter analysis are based on the data expansion of the model without comparison and verification of all the data.

## Figures and Tables

**Table 1:** Pearson correlation analysis.

	*T*_1_	*T*_2_	*H*_2_	HIC
HIC	−0.073	−0.123 **	0.446 **	1

** Correlation is significant at the 0.01 level (2-tailed).

**Table 2:** Classification of braking curves for different inflation masses.

Inflation Mass	*H*_2_	*T*_1_	*T*_2_	HIC *>* 1000 (%)
*Q*_1_	<1.8			23
	>1.8	135–185	160–200	14.8
*Q*_2_	<1.8			3
	>1.8	160–185	160–200	22
*Q*_3_	<1.8			6.67
	>1.8	160–185	180–200	33.33
*Q*_4_	<1.8			10
	>1.8	160–185	180–200	33.33
*Q*_5_	<1.8			6.67
	>1.8	160–185	100–160	16.67

**Table 3:** Graded prediction function.

Inflation Value		*Q*_1_	*Q*_2_	*Q*_3_	*Q*_4_	*Q*_5_
Ratio	H	477.75	703.69	727.85	730.8	697.66
	B_1_	1.46	2.86	−0.3	1.35	4.59
	B_2_	−0.018	−0.074	0.04	−0.03	−0.134
	B_3_	1.37 × 10^4^	6.7 × 10^4^	−4.3 × 10^4^	2.36 × 10^4^	1.14 × 10^4^
Prediction function		HIC(Q) = H + B_1_X + B_2_X^2^ + B_3_X^3^				

## Data Availability

The datasets used and/or analyzed during the current study are available from the corresponding author on reasonable request. All data generated or analyzed during this study are included in this article.
